# Activity of Antiseptics Against *Pseudomonas aeruginosa* and Its Adaptation Potential

**DOI:** 10.3390/antibiotics14010030

**Published:** 2025-01-03

**Authors:** Tomasz M. Karpiński, Marzena Korbecka-Paczkowska, Mark Stasiewicz, Aleksandra E. Mrozikiewicz, Donald Włodkowic, Judyta Cielecka-Piontek

**Affiliations:** 1 Chair and Department of Medical Microbiology, Poznań University of Medical Sciences, Rokietnicka 10, 60-806 Poznań, Poland; mkorbecka@wp.pl (M.K.-P.); markstasiewicz3@gmail.com (M.S.); 2 Medi Pharm, os. Konstytucji 3 Maja 14/2, 63-200 Jarocin, Poland; 3 Department of Internal Medicine, Kirk Kerkorian School of Medicine at UNLV, 1701 W Charleston Blvd Suite 250, Las Vegas, NV 89102, USA; 4 Department of Reproduction, Poznań University of Medical Sciences, Polna 33, 60-535 Poznań, Poland; asm@data.pl; 5 The Neurotox Lab, School of Science, RMIT University, Plenty Road, P.O. Box 71, Bundoora, VIC 3083, Australia; donald.wlodkowic@rmit.edu.au; 6 Department of Pharmacognosy and Biomaterials, Poznań University of Medical Sciences, Rokietnicka 3, 60-806 Poznań, Poland

**Keywords:** clinical concentration, clinical dose, fold change, antimicrobial resistance, AMR, resistance development

## Abstract

Background/Objectives: *Pseudomonas aeruginosa* rapidly acquires antibiotic resistance and demonstrates increasing tolerance to antiseptics. This study evaluated the activity of eight antiseptics against *P. aeruginosa*, assessed its ability to develop adaptation to these antiseptics, and, for the first time, determined the Karpinski Adaptation Index (KAI) for this bacterium. Methods: The minimal inhibitory concentration (MIC), susceptibility to antibiotics, bactericidal time according to EN 1040:2005, adaptation potential, and KAI of *P. aeruginosa* strains were evaluated. Results: The most effective antiseptics against *P. aeruginosa*, based on MIC activity, were octenidine dihydrochloride (OCT; mean MIC 11.3 ± 4.5 µg/mL), polyhexamethylene biguanide (PHMB; MIC 22.6 ± 8.0 µg/mL), and chlorhexidine digluconate (CHX; MIC 26.6 ± 14.4 µg/mL). Sodium hypochlorite (NaOCl) and ethacridine lactate (ET) showed moderate activity, while boric acid (BA), povidone-iodine (PVI), and potassium permanganate (KMnO_4_) exhibited the weakest MIC activity. MIC values for NaOCl (95 ± 15.4 µg/mL) and KMnO_4_ (>10 mg/mL) were close to or exceeded the clinical concentrations used in commercial products. OCT, CHX, and PVI exhibited the fastest bactericidal effect within 1 min. Bactericidal times were up to 15 min for PHMB, up to 60 min for ET, and more than 60 min for BA, NaOCl, and KMnO_4_. The lowest KAI values, indicating a low resistance risk, were observed for OCT (0.12), PHMB (0.19), and BA (0.19). Moderate resistance risk was noted for PVI (0.21), CHX (0.29), and ET (0.47). The highest KAI values, signifying a very high resistance risk, were found for NaOCl (1.0) and KMnO_4_ (≥1.0). Conclusions: Antiseptics like OCT, CHX, and partially PVI can be critical in quick antibacterial action on infected wounds, while agents such as PHMB might be reserved for cases where prolonged contact times are possible. Given the rapid adaptation of *P. aeruginosa* to the clinical concentrations of NaOCl and KMnO_4_ currently in use, reconsideration of their effectiveness in treating skin and mucous membrane infections is recommended.

## 1. Introduction

Currently, we are experiencing a crisis related to antibiotics and the emergence of multidrug-resistant strains. Among the most dangerous are bacteria such as *Klebsiella* sp., *Escherichia* sp., *Acinetobacter* sp., and *Pseudomonas* sp., which possess New Delhi metallo-β-lactamase-1 (NDM-1). These strains are resistant to most antibiotics, including carbapenems [1]. According to the World Health Organization (WHO), the top-priority multidrug-resistant pathogens, collectively referred to as ESKAPE, include *Enterococcus faecium*, *Staphylococcus aureus*, *Klebsiella pneumoniae*, *Acinetobacter baumannii*, *Pseudomonas aeruginosa*, and *Enterobacter* spp. [2].

*Pseudomonas aeruginosa* is an aerobic, motile, rod-shaped, and Gram-negative bacterium. It is commonly isolated from aqueous environments but can also colonize the skin, throat, and stools of a small percentage of individuals [3]. It is an opportunistic pathogen that primarily infects immunocompromised individuals. *P. aeruginosa* is responsible for approximately 20% of hospital-associated infections (HAIs) in Europe and the United States [4] and 4% of surgical site infections in Europe [5]. *P. aeruginosa* is a significant cause of nosocomial (hospital-acquired) infections, including pneumonia (particularly ventilator-associated pneumonia), as well as urinary tract, wound, burn, and bloodstream infections. It also causes community-acquired infections, such as gastrointestinal disorders, skin and wound infections, external otitis, and lower respiratory tract infections in patients with cystic fibrosis [4,6,7].

*P. aeruginosa* produces various virulence factors that influence the development of infections. The major virulence factor is lipopolysaccharide (LPS), a component of the bacterial cell wall. LPS is associated with antibiotic tolerance and biofilm formation. Another important factor are the outer membrane proteins, which play roles in transportation, adhesion, and antibiotic resistance. Drug efficacy is further impaired by the formation of biofilms, alginate-based structures that protect bacteria from external factors. Biofilm formation is a key driver of adaptive resistance development [3,8,9]. *P. aeruginosa* can also produce toxins such as exotoxin A, which inhibits host protein synthesis, and pyocyanin, which damages host tissues. Additionally, it secretes leukocidin, a cytotoxic factor targeting lymphocytes and neutrophils, as well as proteolytic enzymes like elastases and proteases. The bacterium also produces antioxidant enzymes, including catalases, reductases, and superoxide dismutases, which neutralize the activity of phagocytes by reducing reactive oxygen species (ROS) [3,9].

*P. aeruginosa* exhibits intrinsic resistance to many antibiotics, including β-lactams, aminoglycosides, and fluoroquinolones. This resistance is associated with the low permeability of its outer membrane, the expression of efflux pumps, and the production of β-lactamases, enzymes that inactivate antibiotics. Additionally, *P. aeruginosa* strains can acquire resistance through gene transfer and mutations, which can result in resistance to most antibiotics [7,8].

To date, it has been shown that repeated exposure to chlorhexidine or cetylpyridinium chloride leads to an increase in their MIC. This increase was partially retained after subsequent growth without antiseptics [10]. In the case of antiseptics, the potential for bacterial adaptation to antiseptics is known to compounds such as chlorhexidine gluconate [11], benzalkonium chloride [11,12], cetylpyridinium chloride [13], triclosan [14], hydrogen peroxide, and povidone-iodine [15,16]. It is assumed that the development of adaptation to antimicrobial compounds may lead to the emergence of resistance and their lack of clinical effectiveness [17]. This is confirmed by recent studies, which have shown that adaptation to sodium hypochlorite and potassium permanganate can lead to the resistance of *Candida albicans* to the clinical concentrations of these compounds [18]. Some studies have also reported cross-adaptation, resulting in increased MIC of other antiseptics and/or antibiotics [19,20,21]. The suggested mechanism of bacterial adaptation to antiseptics involves an increase in cell surface hydrophobicity and changes in the expression of proteins involved, among others, in membrane transport, virulence, efflux, oxidative stress protection, and metabolism [10,21].

The present work aimed to evaluate the activity of clinical concentrations used in commercial products of antiseptics against *Pseudomonas aeruginosa*. To evaluate whether the adaptation of *P. aeruginosa* to antiseptics observed in the study could pose a risk of resistance development, the Karpinski Adaptation Index (KAI) was applied [22]. This index provides a quantitative assessment of the adaptation level relative to the clinical concentrations of the antiseptics, and its usefulness has been confirmed in previous studies [18].

## 2. Results

### 2.1. Minimal Inhibitory Concentrations (MIC)

OCT demonstrated exceptionally high effectiveness, with MIC values ranging from 7.8 to 15.6 µg/mL, which are significantly lower than its maximum clinical concentration. This antiseptic was effective against all *P. aeruginosa* strains, maintaining a clear safety margin and establishing itself as one of the most potent antiseptics. Similarly, PHMB and CHX exhibited excellent efficacy, with MIC values ranging from 15.6 to 31.25 µg/mL, remaining well below their respective clinical concentrations. These agents exhibit potential for effective action against *P. aeruginosa* with minimal risk of resistance selection.

ET demonstrated higher MIC values (125–250 µg/mL), which, although still below its maximum clinical concentration, require higher doses that may increase the risk of side effects. MIC values for PVI ranged from 4.7 to 18.8 mg/mL, and for BA, from 3.75 to 7.5 mg/mL. Both compounds showed moderate efficacy against *P. aeruginosa*, with PVI’s activity being more variable depending on the strain.

**Figure 1:**
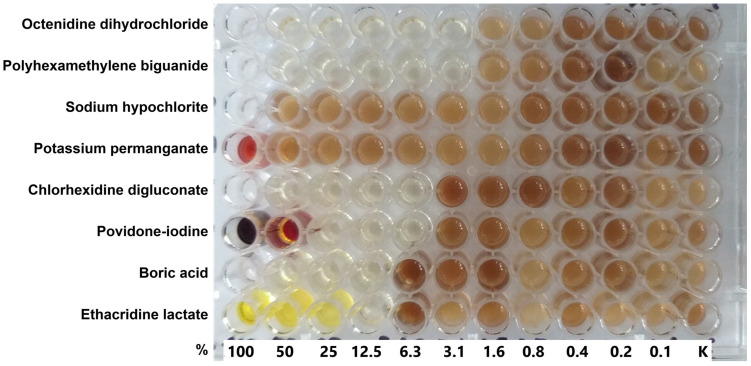
A photo of the minimal inhibitory concentrations (MIC) test for strain 7 of *Pseudomonas aeruginosa*. This strain produces a brown pigment, so the brown coloration indicates bacterial growth. At the same time, the production of pigments, e.g., brown, by *P. aeruginosa* does not seem to be dependent on the concentration of the antiseptic. Some antiseptics at high concentrations also exhibit coloration, such as potassium permanganate, povidone-iodine, and ethacridine lactate. The “%” symbol indicates the percentage concentration of the antiseptic, where 100% corresponds to the clinical concentrations used in commercial products (see Table 1).

In contrast, most of the MIC values for NaOCl were 100 µg/mL, which equals its maximum clinical concentration, indicating no safety margin. This situation could heighten the risk of selecting resistant strains in clinical settings. Similarly, KMnO_4_ demonstrated limited utility, with most strains falling within its maximum clinical concentration range, although some exhibited higher MIC values (>10 mg/mL). These findings suggest that NaOCl and KMnO_4_ have restricted potential for treating *P. aeruginosa*. Detailed data are presented in Table 1, with a sample MIC test image shown in Figure 1.

The MIC values for *P. aeruginosa* strains before and after the adaptation study (Section 2.4) to various antiseptics are presented in Table 2. They show how exposure to these agents affected the susceptibility of the strains, highlighting trends and potential risks of resistance development.

OCT demonstrated consistent MIC values after adaptation. The MICs remained close to the control values regardless of the antiseptic used for adaptation. As observed, adaptation with CHX resulted in a significant cross-increase in MIC levels of OCT, but not exceeding twice the control values. This indicates that OCT maintains its effectiveness against *P. aeruginosa* and poses a minimal risk of resistance development. In contrast, PHMB showed a significant increase in MIC values after adaptation, particularly when the strains were exposed to PHMB itself. The increase is statistically significant, suggesting a potential risk of reduced susceptibility with prolonged or repeated use. CHX showed a moderate increase in MIC values after adaptation to antiseptics and only significant to CHX itself.

For NaOCl, the MIC values increase significantly after adaptation to this agent and KMnO_4_. For KMnO_4_, the MIC values are the same after adaptation to this agent. However, for strains 2, 4, and 8, the MIC values for KMnO_4_ amounted to clinical concentration or above. This lack of a safety margin highlights a high risk of resistance development, especially when either compound is repeatedly used in clinical settings.

PVI exhibited stable MIC values across all conditions, regardless of the adaptation agent. This stability suggests that PVI has a low potential for inducing adaptation. For BA, there is a slight increase in MIC values after adaptation to BA itself. Lastly, ET showed minimal changes in MIC values, with a slight increase after adaptation to specific antiseptics.

### 2.2. Antibiotic Susceptibility Testing

For most antibiotics, including amikacin (AK), aztreonam (ATM), ciprofloxacin (CIP), gentamicin (CN), imipenem (IPM), levofloxacin (LEV), tobramycin (TOB), and piperacillin/tazobactam (TZP), the inhibition zones remained stable, indicating no significant changes in susceptibility after the adaptation study (Section 2.4). Although decreases in inhibition zone diameters were observed for ATM, TOB, and TZP, particularly after adaptation to ET, these changes were not statistically significant.

However, significant reductions in inhibition zones were observed for certain antibiotics after adaptation to specific antiseptics. For ceftazidime (CAZ) and piperacillin (PRL), adaptation to ET significantly decreased the inhibition zones, indicating reduced susceptibility. Similarly, colistin (CT) exhibited notable reductions in inhibition zones following adaptation to BA and ET. Norfloxacin (NOR) showed marked decreases in inhibition zones after adaptation to NaOCl and ET. In contrast, cefepime (FEP) demonstrated no activity against *P. aeruginosa* and showed no significant changes in inhibition zones after adaptation with all the antiseptics tested.

Among the antiseptics tested, ET had the most pronounced cross-effect, causing reductions in inhibition zones for multiple antibiotics, including CAZ, CT, and PRL. These findings underscore that adaptation to certain antiseptics, particularly ET, can impact the susceptibility of *P. aeruginosa* to various antibiotics, potentially increasing the risk of resistance development. The detailed results are presented in Table 3.

### 2.3. Bactericidal Time Study According to EN 1040:2005

To meet the requirements of EN 1040:2005, an antiseptic must demonstrate a 5-log reduction against the target microorganism [23]. This corresponds to reducing the pathogen count by 100,000 times or eliminating 99.999% of microbes or colony-forming units.

**Figure 2:**
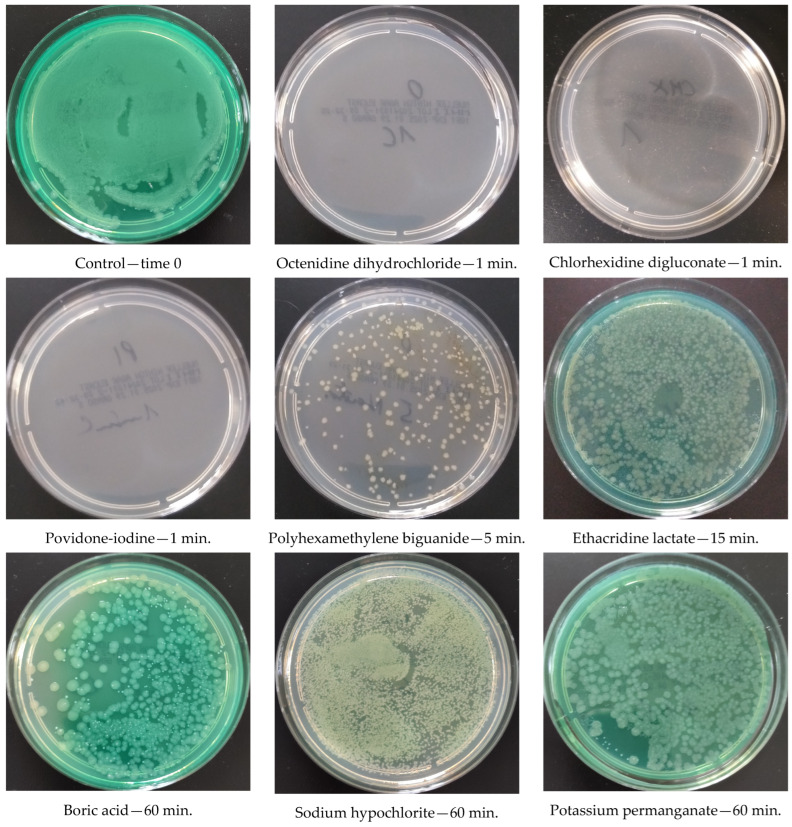
Sample photos of the growth of *Pseudomonas aeruginosa* or the bactericidal effect after incubation with a given antiseptic at clinical concentration for the mentioned time.

Studies showed that OCT, CHX, and PVI reduced the planktonic form of *P. aeruginosa* by over 5-log (log R = 5.379) in under one minute. In comparison, PHMB exhibited insufficient activity within 5 min, achieving a log R of only 4.145. A 15-minute contact time was necessary for PHMB to reach optimal efficacy, whereas ET required up to 60 min to achieve a comparable >5-log reduction. Meanwhile, NaOCl, KMnO_4_, and BA exhibited considerable bacterial growth even after one hour, with a log R of 4.006. The results are presented in Figure 2.

### 2.4. Adaptation of P. aeruginosa Strains to Antiseptics

**Figure 3:**
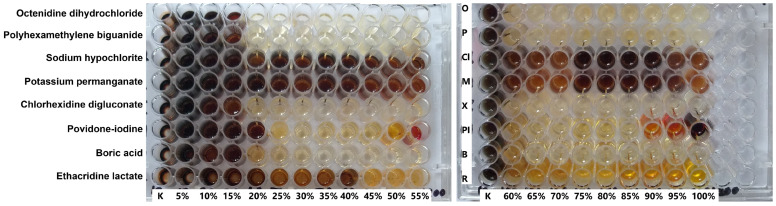
Example image of plates from an adaptation study showing the effect of increasing concentrations of eight antiseptics on *Pseudomonas aeruginosa* strain 7. The antiseptic doses ranged from 5% to 100% of the clinical concentrations used in commercial products, with increments of 5% every two days. Some antiseptics at high concentrations also exhibit coloration, such as povidone-iodine and ethacridine lactate.

The detailed data on the adaptation of *P. aeruginosa* strains to antiseptics is presented in Table 4 and Table 5. An example image of the adaptation study is shown in Figure 3.

The data in Table 4 and Table 5 demonstrate varying levels of adaptation among *P. aeruginosa* strains to different antiseptics, offering valuable insights into their potential risks for resistance development. OCT and PHMB showed significant increases in bacterial tolerance following adaptation, with a maximum fold increase in MIC reaching 12.8. However, the Karpinski Adaptation Index (KAI) values, 0.12 for OCT and 0.19 for PHMB, indicate a low risk of clinical resistance for both antiseptics. These findings suggest that, despite the observed adaptation, these antiseptics maintain a sufficient safety margin, making the likelihood of resistance development by *P. aeruginosa* relatively low.

CHX exhibited a substantial increase in bacterial tolerance after adaptation, with the highest fold increase in MIC reaching 22.4. Its moderate KAI value of 0.29 signals an elevated risk of resistance development. This underscores the critical need for careful monitoring and judicious use of CHX in infection control practices to preserve its long-term efficacy and mitigate the risk of resistance emergence.

BA and PVI demonstrated relatively minor increases in bacterial tolerance, with BA showing a low KAI value of 0.19 and PVI a moderate KAI value of 0.21. These findings suggest that these antiseptics are less likely to drive significant adaptation in *P. aeruginosa*.

ET showed a moderate increase in bacterial tolerance following adaptation, accompanied by a moderate KAI value that amounted to 0.47. This indicates a notable but manageable risk of resistance development, emphasizing the need for careful use of this agent to maintain its clinical utility.

In contrast, NaOCl and KMnO_4_ displayed no significant increase in tolerance during adaptation; however, their high KAI values (1.0 and ≥1.0, respectively) raise concerns. For these agents, the clinical concentrations are close to the observed adaptation thresholds, suggesting a very high risk of resistance development when these antiseptics are repeatedly used in clinical settings.

These findings illustrate the variability in the ability of *P. aeruginosa* to adapt to different antiseptics. They highlight the importance of evaluating both adaptation levels and KAI values to guide the use of antiseptics in clinical and non-clinical environments. Special caution is warranted for antiseptics such as NaOCl, KMnO_4_, and ET, where the risk of resistance development appears to be the highest.

A tabular summary of the obtained MIC results, bactericidal time, and KAI is presented in Table 6.

## 3. Discussion

Antiseptics are used worldwide as a means of infection control and prevention. Recent data indicate that many so-called ‘old antiseptics’, such as KMnO_4_, BA, and ET, should no longer be used today. This is related, among other factors, to their toxicity, side effects, or weak antimicrobial activity [24,25]. In these studies, the weak efficacy of KMnO_4_ against *P. aeruginosa* and a moderate KAI index for ET were confirmed. According to current guidelines in wound lavaseptic and treatment, OCT, PHMB, PVI, or hypochlorites are recommended for use [26,27,28]. In oral antiseptics, CHX is also commonly applied [29,30]. As demonstrated in this article, OCT, PHMB, PVI, and CHX show good activity against *P. aeruginosa*. OCT and PHMB have KAI values at the low-risk level, while PVI and CHX have KAI values at the lower boundary of a moderate risk of resistance development.

Studies have shown that the bactericidal time against planktonic *P. aeruginosa* for OCT, CHX, and PVI is less than 1 min. Similar results for OCT are reported in other publications. Products containing OCT at concentrations of 500 or 1000 µg/mL eliminated *S. aureus* within 15 s of contact time and *S. epidermidis* and *K. pneumoniae* within 40 s [31,32]. Data for CHX and PVI indicate that they have a bactericidal effect against *S. aureus* planktonic bacteria within 15 min but are not bactericidal against biofilm [33]. This is confirmed by in vivo studies conducted by Traore et al. [34], which showed that even 2 h of CHX and PVI application on the skin did not eliminate aerobic and anaerobic microbiota. Similarly, another study [35] demonstrated that 2 h of CHX and PVI exposure to biofilms of *P. aeruginosa* and methicillin-resistant *S. aureus* (MRSA) did not result in complete bacterial eradication. In studies on *Candida* fungi, OCT, CHX, and PVI showed fungicidal activity after 30 min of incubation [36].

In the presented studies, the required bactericidal time for PHMB ranged between 5 and 15 min. In the research by Lu et al. [37], it was found that a 99.99% reduction in the number of *Streptococcus agalactiae*, *S. dysgalactiae*, *S. aureus*, and *E. coli* required 5 min. However, for a product containing 1 mg/mL of PHMB, a 5-log reduction against *S. aureus* was achieved after 60 s of contact time [32].

For NaOCl, KMnO_4_, and BA, it was demonstrated that even after 1 h of incubation, a 5-log reduction of *P. aeruginosa* was not achieved. For KMnO_4_ and ET, no relevant literature data were found describing the speed of the bactericidal effect. In the case of BA, a study was identified indicating that this compound requires 1 h against *Candida albicans* and 2 h against non-albicans *Candida* for a significant 3-log10 reduction [36]. Literature data concerning hypochlorites are varied. Some studies reported rapid antibacterial activity of products containing hypochlorites, including hypochlorous acid and NaOCl at low concentrations, achieving a 3-log or 5-log reduction within 30 s to 3 min [32,38,39]. Research by Wang et al. [40] indicates that hypochlorous acid acts faster, achieving effects within 1 min, whereas NaOCl requires 5 min for *E. coli* and up to 20 min for *P. aeruginosa*. Some studies suggest that NaOCl exhibits antibacterial activity only at very high concentrations. Vianna et al. [41] presented that 0.5% (5 mg/mL) NaOCl required 30 min to completely kill *Enterococcus faecalis*, *S. aureus,* and *C. albicans*. Similarly, other studies found that 0.5% NaOCl eliminated *E. faecalis* after 30 min of incubation [42]. An experiment conducted on infected dentin cylinders showed that 5.25% NaOCl required 40 min of incubation to kill *E. faecalis*, whereas 1.3% and 2.5% NaOCl were ineffective within the same time frame [43].

*P. aeruginosa* has been known to have the ability to adapt to increasing concentrations of antiseptics. This phenomenon has been demonstrated following exposure to low doses of sodium dodecyl sulfate, didecyldimethylammonium chloride, OCT, CHX, and benzalkonium [44,45,46,47,48]. Sublethal concentrations of antiseptics have been shown to induce phenotypic changes that enhance tolerance, with these changes remaining stable even after prolonged periods without exposure to the antiseptic [45]. This has been confirmed through previous studies on *Candida albicans* demonstrating significantly higher MICs for CHX and ET after adaptation than those prior to adaptation [18]. Similar findings have been reported for *Pseudomonas* adaptation to CHX, where repeated exposure resulted in progressively higher MICs [49]. Increased efflux pump expression appears to contribute to the heightened tolerance of Gram-negative bacteria, such as *Pseudomonas*, to CHX and OCT [44,46,50,51]. In the case of PHMB, the possibility of resistance development in this bacterium has been observed [52].

*P. aeruginosa* exhibits several mechanisms that contribute to tolerance or resistance to oxidizing agents, such as NaOCl and hydrogen peroxide. These agents induce the expression of catalases, peroxidases, and superoxide dismutase, which mitigate the effects of oxidative stress [53,54,55]. Exposure to hypochlorites also triggers the expression of chaperones and DNA repair systems while altering membrane permeability through increased hydrophobicity and reduced porin expression. These membrane changes are further associated with the upregulation of efflux pumps and beta-lactamases, leading to enhanced tolerance to chlorine-based biocides and cross-resistance to antibiotics such as ceftazidime, chloramphenicol, and ampicillin [56,57,58]. Additionally, sublethal concentrations of NaOCl have been shown to induce biofilm formation. The production of exopolysaccharides within these biofilms contributes to increased tolerance against oxidative stressors [59,60].

## 4. Materials and Methods

### 4.1. Used Antiseptics and Bacteria

The selection of antiseptics for the study was based on current guidelines of wound treatment [25,26,27,28], which recommend the following four compounds: OCT, PHMB, PVI, and hypochlorites. Additionally, CHX used in oral and wound infections and three widely available antiseptic substances (KMnO_4_, BA, and ET), no longer recommended for wound treatment, were also examined. All 8 antiseptics are listed in Table 7. The initial concentrations of active substances were identical to those found in clinical/commercial products used for wound antiseptics.

This study utilized 10 strains of *P. aeruginosa*, including a reference strain Boston 41501 ATCC-27853 (LGC Standards, Łomianki, Poland) and 9 clinical strains obtained from skin wounds, which are part of the collection of the Department of Medical Microbiology at PUMS. All strains were confirmed on cetrimide agar and through biochemical methods using the ERBA bacterial identification system (Erba Lachema, Brno, Czech Republic).

### 4.2. Minimal Inhibitory Concentrations (MIC)

The minimal inhibitory concentrations (MIC) of antiseptics were determined using the micro-dilution method on 96-well plates (Nest Scientific Biotechnology, Wuxi, China) in tryptic soy broth (TSB; Graso Biotech, Starogard Gdański, Poland). This method is described in detail in our previous publication [30]. Serial dilutions of each antiseptic were prepared, starting from the concentrations specified in Section 2.1. The final bacterial inoculum concentration was 10^5^ CFU/mL. The plates were incubated at 36 °C for 24 h. After incubation, MIC values were determined visually. The tests were performed in 3 repetitions. In interpreting the results, a new index, Clinical Efficiency of MIC (CEMIC), was introduced, representing the ratio of MIC values to clinical concentrations, according to the formula:

$$Clinical\;Efficiency\;of\;MIC\;(CEMIC)\;=\frac{MIC}{Clinical\;concentration}$$

Efficiency was classified as excellent for values < 0.1, moderate for values ranging from 0.1 to 0.9, and poor for levels > 0.9.

### 4.3. Antibiotic Susceptibility Testing

Antibiotic susceptibility testing was performed using the disk diffusion method on Mueller Hinton II Agar, following the EUCAST guidelines (Graso Biotech, Starogard Gdański, Poland). The density of the bacterial inoculum was adjusted to the McFarland 0.5 turbidity standard, which corresponds to approximately 1.5 × 10^8^ CFU/mL. Ready-to-use antibiotic disks with clinical concentrations were applied, included amikacin 30 µg (AK), aztreonam 30 µg (ATM), ceftazidime 30 µg (CAZ), ciprofloxacin 5 µg (CIP), gentamicin 10 µg (CN), colistin sulfate 50 µg (CT), imipenem 10 µg (IPM), levofloxacin 5 µg (LEV), norfloxacin 10 µg (NOR), piperacillin 30 µg (PRL), tobramycin 10 µg (TOB), and piperacillin/tazobactam 36 µg (TZP) (Argenta, Poznań, Poland). The inhibition zones were measured and recorded in millimeters (mm).

### 4.4. Bactericidal Time Study According to EN 1040:2005

The kill-time required for the bactericidal activity of antiseptics was studied in accordance with the European Standard EN 1040:2005 “Chemical disinfectants and antiseptics—Quantitative suspension test for the evaluation of basic bactericidal activity of chemical disinfectants and antiseptics—Test method and requirements (phase 1)” [23]. In this study, a modification was applied, and a reference strain of *P. aeruginosa* Boston 41501 ATCC-27853 (strain no. 1) was used. In brief, 1 mL of water was mixed with 1 mL of bacterial cell suspension at a density of 1.5 × 10^8^ CFU/mL and 8 mL of antiseptics. The activity of clinically used concentrations and the control MICs of antiseptics were evaluated. The mixture was incubated for 5, 15, and 60 min at room temperature. After each incubation period, 1 mL of the sample was transferred to a neutralizer and left at room temperature for 5 min to neutralize the antiseptic’s activity. Subsequently, 1 mL of each sample was inoculated onto agar, and the Petri dishes were incubated at 37 °C for 24 h. The results were interpreted following the guidelines of EN 1040:2005.

### 4.5. Adaptation of P. aeruginosa Strains to Antiseptics

**Figure 4:**
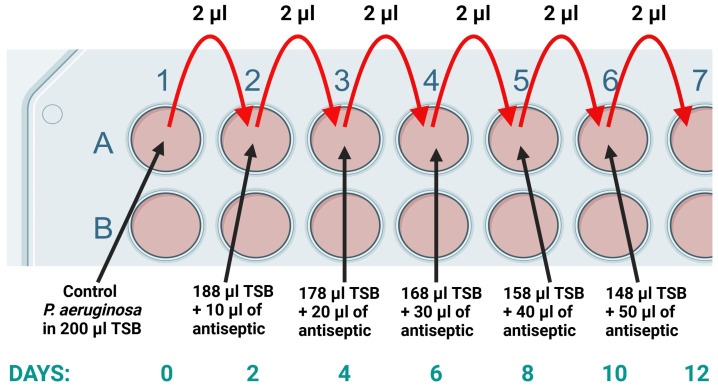
The initial stages of the study on the adaptation of *Pseudomonas aeruginosa* strains to antiseptics were conducted using 96-well plates. In this setup, the bacteria were cultured in tryptic soy broth (TSB) containing a starting antiseptic concentration equal to 5% of the clinical concentrations used in commercial formulations. This low concentration allowed the bacteria to survive and initiate adaptation without immediate inhibition.

*P. aeruginosa* strains were initially cultured at 5% of the clinical concentrations of each antiseptic used in commercial products, equivalent to 25 µg/mL OCT, 50 µg/mL PHMB, 5 µg/mL NaOCl, 0.5 mg/mL KMnO_4_, 50 µg/mL CHX, 3.75 mg/mL PVI, 1.5 mg/mL BA, and 50 µg/mL ET. Additionally, for OCT, PHMB, and CHX, testing was performed at concentrations ranging from 0.5% to 4.5% of the initial clinical concentration, which corresponded to 2.5 µg/mL OCT, 5 µg/mL PHMB, and 5 µg/mL CHX (Figure 4).

The experiments were conducted according to details in our previous publications [18,22]. The 96-well plates with 200 µL of TSB in each well were applied. Every 48 h, 2 µL of bacterial culture was transferred into new wells filled with TSB, where the concentration of the antiseptic was incrementally increased. This stepwise exposure allowed the bacteria to gradually adapt to higher levels of the antiseptic. The adaptation process was continued systematically until the concentrations reached the full levels of clinical concentrations used in commercial products specified for each antiseptic, as illustrated in Figure 4. The tests were performed in 3 repetitions.

Following the completion of the adaptation phase, the bacteria were passaged twice in TSB without any antiseptic to ensure colony stabilization and to eliminate any transient physiological changes due to direct antiseptic exposure. Adapted colonies underwent additional tests, including a new determination of their MIC values to assess any changes in tolerance to the antiseptics. Furthermore, the antibiotic resistance profiles of these adapted strains were re-examined to evaluate potential cross-resistance or altered susceptibility to commonly used antibiotics.

### 4.6. Karpinski Adaptation Index (KAI)

The KAI for antiseptics can be calculated according to the formula [22]:

$$Karpinski\;Adaptation\;Index\;(KAI)\;=\frac{Adaptation}{Clinical\;concentration}$$

In this paper, “Adaptation” represents the highest concentration of the antiseptic at which the microorganism is still able to grow. Meanwhile, “Clinical concentration” refers to either the standard commercially available concentration, the therapeutic dose used to treat a particular disease, or the recommended dosage for the compound. In summary, KAI values of ≤0.1 indicate a very low risk of clinical resistance, as the level of bacterial adaptation remains significantly below the antiseptic’s clinical concentration. Values between >0.1 and ≤0.2 represent a low risk of resistance development, while values ranging from >0.2 to ≤0.8 suggest a moderate risk. A KAI value > 0.8 but <1.0 indicates a high risk of clinical resistance. Finally, a KAI value of ≥1.0 signals a very high risk of resistance or suggests that resistance may have already developed.

### 4.7. Statistics

One-way ANOVA with Tukey post-tests were applied to determine the statistical significance of differences in the biofilm bacteria numbers. The results were considered significant at the level of *p* < 0.05. Data were tested using InStat v3.10 software (GraphPad Software, Boston, MA, USA).

## 5. Conclusions

The best activity against *P. aeruginosa* was demonstrated by OCT, PVI, and CHX, which exhibited the fastest bactericidal effect within 1 min. This rapid action underscores their effectiveness in quickly reducing bacterial populations, which is particularly advantageous in clinical settings where time-sensitive interventions are crucial. PHMB showed effective bactericidal activity within 15 min, making it a slower but still reliable agent in controlling *P. aeruginosa*. In contrast, other agents required significantly more time to achieve similar reductions, indicating their limited efficiency.

Additionally, OCT and PHMB had low KAI values, signifying a minimal risk of resistance development. This combination of rapid bactericidal action and low resistance potential highlights their suitability as first-line choices for treating infections caused by *P. aeruginosa*. ET, PVI, and BA exhibited weaker activity, with KAI values for PVI and BA falling between low and moderate risk, while ET indicated a moderate risk of clinical resistance development. The poorest activity and highest KAI values were observed for NaOCl and KMnO_4_, which not only demonstrated a prolonged bactericidal effect but also allowed significant bacterial survival even after extended exposure. These findings highlight a substantial risk of resistance development, especially with repeated clinical use of these agents, necessitating caution and the exploration of alternative solutions.

To summarize, antiseptics like OCT, CHX, and partially PVI can be critical in quick antibacterial action on infected wounds, while agents such as PHMB might be reserved for cases where prolonged contact times are possible.

## Figures and Tables

**Table 1:** Minimal inhibitory concentration (MIC) values and Clinical Efficiency of MIC (CEMIC) of the tested antiseptics against *Pseudomonas aeruginosa* before adaptation studies.

Antiseptic	Clinical Concentration	MIC Values of *P. aeruginosa* Strains Numbered Below										Clinical Efficiency of MIC (CEMIC)
		1 (Boston)	2	3	4	5	6	7	8	9	10	
OCT (µg/mL)	500	7.8	3.9	7.8–15.6	15.6	7.8	15.6	15.6	15.6	7.8	7.8–15.6	Excellent
PHMB (µg/mL)	1000	31.25	15.6	15.6	15.6	31.25	15.6	31.25	15.6	15.6–31.25	31.25	Excellent
CHX (µg/mL)	1000	31.25	15.6	15.6	31.25	15.6	31.25	62.5	31.25	15.6	15.6	Excellent
NaOCl (µg/mL)	100	100	100	100	100	50	100	100	100	100	100	Poor
KMnO_4_ (mg/mL)	10	10	>10	10	>10	10	10	10	>10	10	10	Poor
PVI (mg/mL)	75	4.7	18.8	4.7	4.7	4.7	4.7	4.7–9.4	4.7	4.7	4.7	Moderate
BA (mg/mL)	30	3.75	3.75	7.5	3.75	3.75	3.75	3.75	3.75	7.5	3.75	Moderate
ET (µg/mL)	1000	125	250	125	250	250	125	125	250	250	250	Moderate

CEMIC was classified as excellent for values < 0.1, moderate for values between 0.1 and 0.9, and poor for values > 0.9. Abbreviations: OCT—octenidine dihydrochloride, PHMB—polyhexamethylene biguanide, CHX—chlorhexidine digluconate, NaOCl—sodium hypochlorite, KMnO_4_—potassium permanganate, PVI—povidone-iodine, BA—boric acid, and ET—ethacridine lactate.

**Table 2:** Minimum inhibitory concentration (MIC) values for control samples before adaptation and for samples after adaptation with a specific antiseptic against 10 *Pseudomonas aeruginosa* strains.

Antiseptic (Units)	Control MICs Before Adaptation	MIC Values (Mean ± SD) After Adaptation with Below Antiseptic							
		OCT	PHMB	NaOCl	KMnO_4_	CHX	PVI	BA	ET
OCT (µg/mL)	11.3 ± 4.5 (7.9–15.6)	13.6 ± 5.6 (7.8–31.25)	14.4 ± 5.2 * (7.8–15.6)	13.7 ± 3.5 (7.8–31.25)	14.4 ± 5.2 (7.8–31.25)	17.2 ± 7.9 *** (7.8–31.25)	13.6 ± 5.6 (7.8–31.25)	13.6 ± 5.6 (7.8–15.6)	13.3 ± 3.7 (7.8–31.25)
PHMB (µg/mL)	22.6 ± 8.0 (15.6–31.25)	41.4 ± 18.5 (31.25–62.5)	62.5 ± 35.1 ** (31.25–125)	59.4 ± 36.4 * (31.25–125)	59.4 ± 36.4 * (31.25–125)	65.6 ± 41.7 ** (31.25–125)	59.4 ± 36.4 * (31.25–125)	59.4 ± 36.4 * (31.25–125)	59.4 ± 36.4 * (15.6–125)
CHX (µg/mL)	26.6 ± 14.4 (15.6–62.5)	39.8 ± 15.6 (15.6–62.5)	39.8 ± 15.6 (15.6–62.5)	41.4 ± 16.3 (15.6–62.5)	41.4 ± 16.3 (15.6–125)	49.2 ± 24.5 ** (15.6–62.5)	36.7 ± 18.5 (15.6–62.5)	36.7 ± 18.5 (15.6–62.5)	38.3 ± 19.3 (15.6–125)
NaOCl (µg/mL)	95 ± 15.4 (50–100)	100 ± 0.0 (100)	100 ± 0.0 (100)	>100 *** (100 and >100)	>100 *** (100 and >100)	100 ± 0.0 (100)	100 ± 0.0 (100)	100 ± 0.0 (100)	100 ± 0.0 (100)
KMnO_4_ (mg/mL)	>10 (100 and >100)	>10 (100 and >100)	>10 (100 and >100)	>10 (100 and >100)	>10 (100 and >100)	>10 (100 and >100)	>10 (100 and >100)	>10 (100 and >100)	>10 (100 and >100)
PVI (mg/mL)	6.8 ± 4.4 (4.7–18.8)	8.2 ± 5.7 (4.7–18.8)	8.2 ± 5.7 (4.7–18.8)	8.2 ± 5.7 (4.7–18.8)	8.2 ± 5.7 (4.7–18.8)	8.2 ± 5.7 (4.7–18.8)	8.2 ± 5.7 (4.7–18.8)	8.2 ± 5.7 (4.7–18.8)	8.2 ± 5.7 (4.7–18.8)
BA (mg/mL)	4.5 ± 1.5 (3.75–7.5)	4.5 ± 1.5 (3.75–7.5)	4.5 ± 1.5 (3.75–7.5)	4.5 ± 1.5 (3.75–7.5)	5.3 ± 3.5 (3.75–15)	4.5 ± 1.5 (3.75–7.5)	4.5 ± 1.5 (3.75–7.5)	7.3 ± 5.2 * (3.75–15)	4.5 ± 1.5 (3.75–7.5)
ET (µg/mL)	200 ± 62.8 (125–250)	212.5 ± 58.8 (125–250)	212.5 ± 58.8 (125–250)	212.5 ± 58.8 (125–250)	212.5 ± 58.8 (125–250)	212.5 ± 58.8 (125–250)	212.5 ± 58.8 (125–250)	212.5 ± 58.8 (125–500)	225 ± 87.0 (125–500)

Statistically significant differences compared to the control before adaptation: * *p* < 0.05, ** *p* < 0.01, *** *p* < 0.001. Abbreviations: OCT—octenidine dihydrochloride, PHMB—polyhexamethylene biguanide, CHX—chlorhexidine digluconate, NaOCl—sodium hypochlorite, KMnO_4_—potassium permanganate, PVI—povidone-iodine, BA—boric acid, and ET—ethacridine lactate.

**Table 3:** Zones of growth inhibition (ZOI, in mm) for *Pseudomonas aeruginosa* strains before adaptation studies (control) and after adaptation to specific antiseptics.

Antibiotic	Control ZOI Before Adaptation	ZOI After Adaptation with Below Antiseptic (Ranges)							
		OCT	PHMB	NaOCl	KMnO_4_	CHX	PVI	BA	ET
AK	16.8 ± 1.0 (15–18)	17.1 ± 2.2 (15–22)	16.4 ± 1.1 (15–18)	16.3 ± 0.9 (15–18)	16.4 ± 1.8 (14–20)	16.1 ± 1.2 (15–18)	16.4 ± 1.9 (14–20)	17.1 ± 2.0 (15–20)	17.1 ± 0.8 (16–18)
ATM	17.9 ± 6.1 (6–24)	18.5 ± 6.0 (6–24)	17.8 ± 6.6 (6–24)	16.0 ± 6.4 (6–22)	13.1 ± 8.0 (6–23)	15.8 ± 6.6 (6–22)	15.1 ± 6.0 (6–21)	17.0 ± 5.7 (6–24)	13.4 ± 6.4 (6–20)
CAZ	18.8 ± 6.3 (6–24)	17.9 ± 7.0 (6–25)	16.0 ± 6.1 (6–22)	16.9 ± 6.5 (6–24)	17.4 ± 6.7 (6–25)	17.3 ± 7.4 (6–25)	16.3 ± 6.2 (6–22)	18.1 ± 6.4 (6–25)	8.3 ± 4.2 *** (6–16)
CIP	25.3 ± 8.2 (6–32)	24.9 ± 7.8 (6–30)	25.1 ± 8.0 (6–30)	25.3 ± 8.4 (6–32)	24.4 ± 8.1 (6–32)	23.4 ± 7.1 (6–27)	24.3 ± 7.8 (6–30)	23.6 ± 7.6 (6–30)	23.0 ± 7.1 (6–28)
CN	12.9 ± 2.3 (10–17)	15.5 ± 4.1 (12–22)	12.0 ± 3.1 (10–18)	13.4 ± 1.5 (12–16)	12.5 ± 2.4 (9–15)	12.8 ± 1.6 (11–16)	12.6 ± 1.6 (11–15)	14.0 ± 0.9 (13–15)	14.3 ± 0.5 (14–15)
CT	17.3 ± 1.0 (16–19)	16.4 ± 0.5 (16–17)	17.6 ± 1.6 (16–20)	17.0 ± 1.6 (15–20)	16.1 ± 0.8 (15–17)	15.8 ± 1.5 (14–18)	16.6 ± 0.5 (16–17)	15.3 ± 0.7 * (14–16)	13.5 ± 1.7 *** (12–16)
FEP	6.0 ± 0.0 (6)	6.0 ± 0.0 (6)	6.0 ± 0.0 (6)	6.0 ± 0.0 (6)	6.0 ± 0.0 (6)	6.0 ± 0.0 (6)	6.0 ± 0.0 (6)	6.0 ± 0.0 (6)	6.0 ± 0.0 (6)
IPM	16.8 ± 4.4 (6–19)	18.3 ± 5.0 (6–20)	17.0 ± 4.9 (6–20)	16.5 ± 4.5 (6–20)	17.3 ± 4.9 (6–20)	16.3 ± 4.4 (6–20)	16.3 ± 4.2 (6–18)	17.8 ± 5.0 (6–22)	17.4 ± 4.8 (6–20)
LEV	23.1 ± 2.0 (20–26)	22.5 ± 3.0 (16–26)	22.6 ± 1.8 (20–25)	22.4 ± 2.1 (20–26)	23.3 ± 4.3 (18–30)	23.4 ± 2.1 (20–26)	22.9 ± 2.1 (20–26)	23.0 ± 1.8 (20–25)	23.4 ± 1.1 (22–25)
NOR	27.4 ± 6.7 (16–36)	26.9 ± 2.2 (25–30)	25.0 ± 4.6 (15–30)	20.3 ± 2.8 * (16–25)	22.4 ± 3.3 (16–25)	22.9 ± 2.8 (16–24)	21.1 ± 2.7 (16–24)	19.4 ± 1.5 ** (16–21)	21.3 ± 6.0 (16–30)
PRL	18.9 ± 3.0 (15–22)	19.0 ± 2.8 (16–22)	13.3 ± 6.4 (6–22)	17.5 ± 7.8 (6–25)	18.6 ± 5.5 (11–24)	16.1 ± 2.6 (14–22)	17.0 ± 2.4 (15–22)	15.1 ± 3.2 (12–22)	8.3 ± 6.4 ** (6–24)
TOB	15.0 ± 3.7 (6–17)	15.3 ± 3.8 (6–18)	14.4 ± 3.5 (6–17)	15.4 ± 3.9 (6–18)	15.0 ± 3.7 (6–17)	15.3 ± 3.9 (6–18)	15.1 ± 3.8 (6–18)	15.8 ± 4.3 (6–20)	10.9 ± 5.2 (6–16)
TZP	18.3 ± 5.5 (6–23)	18.0 ± 5.4 (6–23)	12.5 ± 7.0 (6–20)	18.4 ± 5.9 (6–24)	19.3 ± 6.5 (6–26)	16.6 ± 4.4 (6–19)	14.9 ± 3.8 (6–18)	18.1 ± 4.9 (6–20)	10.0 ± 7.1 (6–22)

Statistically significant differences compared to the control before adaptation: * *p* < 0.05, ** *p* < 0.01, *** *p* < 0.001. Abbreviations: AK—amikacin, ATM—aztreonam, CAZ—ceftazidime, CIP—ciprofloxacin, CN—gentamicin, CT—colistin sulfate, FEP—cefepime, IPM—imipenem, LEV—levofloxacin, NOR—norfloxacin, PRL—piperacillin, TOB—tobramycin, and TZP—piperacillin/tazobactam.

**Table 4:** Adaptation of *Pseudomonas aeruginosa* strains to antiseptics. The highest concentrations of antiseptics at which bacterial growth was observed are shown.

Antiseptic	Clinical Concentration	Adaptation of *Pseudomonas aeruginosa* Strains Numbered Below									
		1 (Boston)	2	3	4	5	6	7	8	9	10
OCT (µg/mL)	500	75	50	75	50	75	50	75	50	50	50
PHMB (µg/mL)	1000	250	150	200	150–200	250	200	150	200	200	150
CHX (µg/mL)	1000	350	250	150–250	450	350	250–350	150	250	350	250
NaOCl (µg/mL)	100	100	100	100	100	100	100	100	100	100	100
KMnO_4_ (mg/mL)	10	10	10	10	10	10	10	10	10	10	10
PVI (mg/mL)	75	15	15	15	18.8	15	15	15	15	15	15
BA (mg/mL)	30	4.5	6	4.5–6	6	6	6	4.5	6	6	6
ET (µg/mL)	1000	450	500	400–450	500	450	550	400	450	500	450

Abbreviations: OCT—octenidine dihydrochloride, PHMB—polyhexamethylene biguanide, NaOCl—sodium hypochlorite, KMnO_4_—potassium permanganate, CHX—chlorhexidine digluconate, PVI—povidone-iodine, BA—boric acid, and ET—ethacridine lactate.

**Table 5:** Fold increase in adaptation to antiseptics relative to the initial MIC and Karpinski Adaptation Index (KAI) of *Pseudomonas aeruginosa* strains.

Antiseptic	Clinical Concentration	Mean Initial MIC ± SD (Before Adaptation)	Mean Adaptation ± SD	Fold Increase in Adaptation Relative to Initial MIC	Karpinski Adaptation Index (KAI)
OCT (µg/mL)	500	11.3 ± 4.5	60 ± 12.6	×3.2–12.8	0.12
PHMB (µg/mL)	1000	22.6 ± 8.0	193 ± 37.3	×4.8–12.8	0.19
BA (mg/mL)	30	4.5 ± 1.5	5.6 ± 0.7	×0.6–1.6	0.19
PVI (mg/mL)	75	6.8 ± 4.4	15.4 ± 1.2	×0.8–4.0	0.21
CHX (µg/mL)	1000	26.6 ± 14.4	290 ± 88.3	×2.4–22.4	0.29
ET (µg/mL)	1000	200 ± 62.8	468 ± 43.8	×1.8–4.4	0.47
NaOCl (µg/mL)	100	95 ± 15.4	100 ± 0.0	×1.0	1.0
KMnO_4_ (mg/mL)	10	10 ± 0.0	10 ± 0.0	×1.0 or <1.0	≥1.0

Interpretation of the adaptation index (KAI): 0.1 < KAI ≤ 0.2: low risk of clinical resistance; 0.2 < KAI ≤ 0.8: moderate risk of clinical resistance; 0.8 < KAI < 1.0: high risk of clinical resistance; KAI ≥ 1.0: very high risk of clinical resistance [22].

**Table 6:** Summary of the activity of eight antiseptics against *Pseudomonas aeruginosa*.

	OCT	CHX	PHMB	PVI	ET	BA	NaOCl	KMnO_4_
Clinical Efficiency of MIC (CEMIC)	Excellent	Excellent	Excellent	Moderate	Moderate	Moderate	Poor	Poor
Bactericidal time (EN 1040:2005)	<1 min.	<1 min.	<15 min.	<1 min.	<60 min.	>60 min.	>60 min.	>60 min.
Karpinski Adaptation Index (KAI)	Low	Moderate	Low	Moderate	Moderate	Low	High	Very high
Recommended use in the treatment of wounds infected with *P. aeruginosa*	Yes	Yes	Yes	Partially	No	No	No	No

**Table 7:** Antiseptics along with their initial concentrations used in the experiments and chemical structure.

Antiseptic and Its Clinical Concentration	Chemical Structure	Producer
Octenidine dihydrochloride, OCT; 500 µg/mL	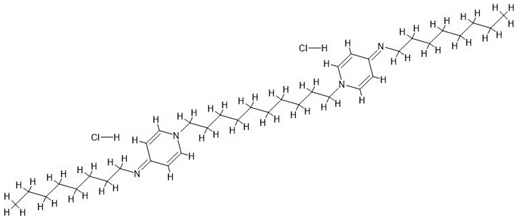	Schülke & Mayr GmbH, Norderstedt, Germany
Polyhexamethylene biguanide, PHMB; 1000 µg/mL	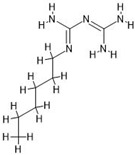	Arxada AG, Basel, Switzerland
Sodium hypochlorite, NaOCl; 100 µg/mL	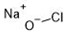	Cerkamed, Stalowa Wola, Poland
Potassium permanganate, KMnO_4_; 10 mg/mL	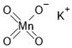	Hasco-Lek S.A., Wrocław, Poland
Chlorhexidine digluconate, CHX; 1000 µg/mL	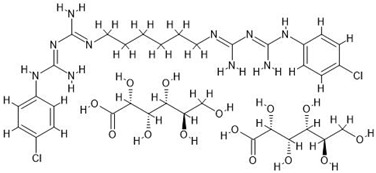	Sigma-Aldrich, Poznań, Poland
Povidone-iodine, PVI; 75 mg/mL	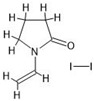	Sigma-Aldrich, Poznań, Poland
Boric acid, BA; 30 mg/mL	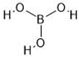	Herbapol, Poznań, Poland
Ethacridine lactate, ET; 1000 µg/mL	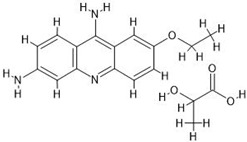	Herbapol, Poznań, Poland

## Data Availability

The original contributions presented in the study are included in the article, further inquiries can be directed to the corresponding author.

## References

[1] Yao S., Yu J., Zhang T., Xie J., Yan C., Ni X., Guo B., Cui C. (2024). Comprehensive Analysis of Distribution Characteristics and Horizontal Gene Transfer Elements of blaNDM-1-Carrying Bacteria. Sci. Total Environ..

[2] Aggarwal R., Mahajan P., Pandiya S., Bajaj A., Verma S.K., Yadav P., Kharat A.S., Khan A.U., Dua M., Johri A.K. (2024). Antibiotic Resistance: A Global Crisis, Problems and Solutions. Crit. Rev. Microbiol..

[3] Qin S., Xiao W., Zhou C., Pu Q., Deng X., Lan L., Liang H., Song X., Wu M. (2022). *Pseudomonas aeruginosa*: Pathogenesis, Virulence Factors, Antibiotic Resistance, Interaction with Host, Technology Advances and Emerging Therapeutics. Signal Transduct. Target. Ther..

[4] Sathe N., Beech P., Croft L., Suphioglu C., Kapat A., Athan E. (2023). *Pseudomonas aeruginosa*: Infections and Novel Approaches to Treatment “Knowing the Enemy” the Threat of *Pseudomonas aeruginosa* and Exploring Novel Approaches to Treatment. Infect. Med..

[5] European Centre for Disease Prevention and Control (2023). Healthcare-Associated Infections: Surgical Site Infections. Annual Epidemiological Report for 2018–2020.

[6] Phan S., Feng C.H., Huang R., Lee Z.X., Moua Y., Phung O.J., Lenhard J.R. (2023). Relative Abundance and Detection of *Pseudomonas aeruginosa* from Chronic Wound Infections Globally. Microorganisms.

[7] Elfadadny A., Ragab R.F., AlHarbi M., Badshah F., Ibáñez-Arancibia E., Farag A., Hendawy A.O., De los Ríos-Escalante P.R., Aboubakr M., Zakai S.A. (2024). Antimicrobial Resistance of *Pseudomonas aeruginosa*: Navigating Clinical Impacts, Current Resistance Trends, and Innovations in Breaking Therapies. Front. Microbiol..

[8] Giovagnorio F., De Vito A., Madeddu G., Parisi S.G., Geremia N. (2023). Resistance in *Pseudomonas aeruginosa*: A Narrative Review of Antibiogram Interpretation and Emerging Treatments. Antibiotics.

[9] Tuon F.F., Dantas L.R., Suss P.H., Tasca Ribeiro V.S. (2022). Pathogenesis of the *Pseudomonas aeruginosa* Biofilm: A Review. Pathogens.

[10] Verspecht T., Rodriguez Herrero E., Khodaparast L., Khodaparast L., Boon N., Bernaerts K., Quirynen M., Teughels W. (2019). Development of Antiseptic Adaptation and Cross-Adapatation in Selected Oral Pathogens in Vitro. Sci. Rep..

[11] Liu W.J., Fu L., Huang M., Zhang J.P., Wu Y., Zhou Y.S., Zeng J., Wang G.X. (2017). Frequency of Antiseptic Resistance Genes and Reduced Susceptibility to Biocides in Carbapenem-Resistant *Acinetobacter baumannii*. J. Med. Microbiol..

[12] El Sayed Zaki M., Bastawy S., Montasser K. (2019). Molecular Study of Resistance of *Staphylococcus aureus* to Antiseptic Quaternary Ammonium Compounds. J. Glob. Antimicrob. Resist..

[13] Mao X., Auer D.L., Buchalla W., Hiller K.-A., Maisch T., Hellwig E., Al-Ahmad A., Cieplik F. (2020). Cetylpyridinium Chloride: Mechanism of Action, Antimicrobial Efficacy in Biofilms, and Potential Risks of Resistance. Antimicrob. Agents Chemother..

[14] Bayston R., Ashraf W., Smith T. (2007). Triclosan Resistance in Methicillin-Resistant *Staphylococcus aureus* Expressed as Small Colony Variants: A Novel Mode of Evasion of Susceptibility to Antiseptics. J. Antimicrob. Chemother..

[15] Merchel Piovesan Pereira B., Wang X., Tagkopoulos I. (2021). Biocide-Induced Emergence of Antibiotic Resistance in *Escherichia coli*. Front. Microbiol..

[16] Williamson D.A., Carter G.P., Howden B.P. (2017). Current and Emerging Topical Antibacterials and Antiseptics: Agents, Action, and Resistance Patterns. Clin. Microbiol. Rev..

[17] Windels E.M., den Bergh B.V., Michiels J. (2020). Bacteria under Antibiotic Attack: Different Strategies for Evolutionary Adaptation. PLOS Pathog..

[18] Karpiński T.M., Korbecka-Paczkowska M., Ożarowski M., Włodkowic D., Wyganowska M.L., Seremak-Mrozikiewicz A., Cielecka-Piontek J. (2024). Adaptation to Sodium Hypochlorite and Potassium Permanganate May Lead to Their Ineffectiveness Against *Candida albicans*. Pharmaceuticals.

[19] Bartsch S., Kohnert E., Kreutz C., Woelber J.P., Anderson A., Burkhardt A.-S., Hellwig E., Buchalla W., Hiller K.-A., Ratka-Krueger P. (2024). Chlorhexidine Digluconate Mouthwash Alters the Oral Microbial Composition and Affects the Prevalence of Antimicrobial Resistance Genes. Front. Microbiol..

[20] Früh R., Anderson A., Cieplik F., Hellwig E., Wittmer A., Vach K., Al-Ahmad A. (2022). Antibiotic Resistance of Selected Bacteria after Treatment of the Supragingival Biofilm with Subinhibitory Chlorhexidine Concentrations. Antibiotics.

[21] Wand M.E., Sutton J.M. (2022). Efflux-Mediated Tolerance to Cationic Biocides, a Cause for Concern?. Microbiology.

[22] Karpiński T.M. (2024). Adaptation Index (KAI)—A New Indicator of Adaptation and Potential Antimicrobial Resistance. Herba Pol..

[23] European Standard EN 1040:2005, PN-EN 1040:2006 “Chemical Disinfectants and Antiseptics—Quantitative Suspension Test for the Evaluation of Basic Bactericidal Activity of Chemical Disinfectants and Antiseptics—Test Method and Requirements (Phase 1)”. https://sklep.pkn.pl/pn-en-1040-2006e.html.

[24] Babalska Z.Ł., Korbecka-Paczkowska M., Karpiński T.M. (2021). Wound Antiseptics and European Guidelines for Antiseptic Application in Wound Treatment. Pharmaceuticals.

[25] Sopata M., Jawień A., Mrozikiewicz-Rakowska B., Augusewicz Z., Bakowska M., Samson I., Gabriel M., Grzela T., Karpiński T., Kuberka I. (2020). Guidelines for Topical Management in Non-Infected, at Risk of Infection and Infected Wounds—An Overview of Available Antimicrobial Substances Used in the Treatment of Wounds. Recommendations of the Polish Wound Treatment Society. Leczenie Ran.

[26] Sopata M., Mrozikiewicz-Rakowska B., Jawień A., Woroń J., Malka M., Karpiński T.M., Sobieszek-Kundro A., Gabriel M., Mańkowski P., Szewczyk M. (2023). Statement of the Polish Wound Management Association—Antimicrobial management in colonized wounds, with signs of infection and at risk of infection in the era of antibiotic resistance. Leczenie Ran.

[27] Nair H.K.R., Mrozikiewicz-Rakowska B., Pinto D.S., Stuermer E.K., Matiasek J., Sander J., Lázaro-Martínez J.L., Ousey K., Assadian O., Kim P.J. (2023). Use of Wound Antiseptics in Practice. International Consensus Document.

[28] Kramer A., Dissemond J., Kim S., Willy C., Mayer D., Papke R., Tuchmann F., Assadian O. (2018). Consensus on Wound Antisepsis: Update 2018. Skin Pharmacol. Physiol..

[29] Garrido L., Lyra P., Rodrigues J., Viana J., Mendes J.J., Barroso H. (2023). Revisiting Oral Antiseptics, Microorganism Targets and Effectiveness. J. Pers. Med..

[30] Korbecka-Paczkowska M., Karpiński T.M. (2024). In Vitro Assessment of Antifungal and Antibiofilm Efficacy of Commercial Mouthwashes against *Candida albicans*. Antibiotics.

[31] Engelhart S., Exner M., Simon A. (2015). In Vitro Study on the Disinfectability of Two Split-Septum Needle-Free Connection Devices Using Different Disinfection Procedures. GMS Hyg. Infect. Control.

[32] Yap J.W., Ismail N.I., Lee C.S., Oh D.Y. (2024). Impact of Interfering Substances on the Bactericidal Efficacy of Different Commercially Available Hypochlorous Acid-Based Wound Irrigation Solutions Commonly Found in South-East Asia. Antibiotics.

[33] Parker D.M., Koch J.A., Gish C.G., Brothers K.M., Li W., Gilbertie J., Rowe S.E., Conlon B.P., Byrapogu V.K.C., Urish K.L. (2023). Hydrogen Peroxide, Povidone-Iodine and Chlorhexidine Fail to Eradicate *Staphylococcus aureus* Biofilm from Infected Implant Materials. Life.

[34] Traoré O., Allaert F.A., Fournet-Fayard S., Verrière J.L., Laveran H. (2000). Comparison of In-Vivo Antibacterial Activity of Two Skin Disinfection Procedures for Insertion of Peripheral Catheters: Povidone Iodine versus Chlorhexidine. J. Hosp. Infect..

[35] Hill D.L., Castiaux A., Pensler E., Knue J., Attar P.S., Siddiqi A. (2022). A Novel Activated Zinc Solution with Improved Efficacy Against *Pseudomonas* and MRSA Biofilm Compared to Chlorhexidine and Povidone-Iodine. Surg. Technol. Int..

[36] Hacioglu M., Oyardi O., Yilmaz F.N., Nagl M. (2022). Comparative Fungicidal Activities of N-Chlorotaurine and Conventional Antiseptics against *Candida* Spp. Isolated from Vulvovaginal Candidiasis. J. Fungi.

[37] Lu Y., Wang D., Zhang Y., Hu Y., Lu J., Zeng Z., Zeng D. (2023). Preparation and Antimicrobial Activity of a Film-Forming Polyhexamethylene Biguanide Teat Disinfectant. Int. J. Mol. Sci..

[38] Velazquez-Meza M.E., Hernández-Salgado M., Sánchez-Alemán M.A. (2015). Evaluation of the Antimicrobial Activity of a Super Oxidized Solution in Clinical Isolates. Microb. Drug Resist..

[39] Landa-Solis C., González-Espinosa D., Guzmán-Soriano B., Snyder M., Reyes-Terán G., Torres K., Gutierrez A.A. (2005). Microcyn: A Novel Super-Oxidized Water with Neutral pH and Disinfectant Activity. J. Hosp. Infect..

[40] Wang L., Bassiri M., Najafi R., Najafi K., Yang J., Khosrovi B., Hwong W., Barati E., Belisle B., Celeri C. (2007). Hypochlorous Acid as a Potential Wound Care Agent: Part I. Stabilized Hypochlorous Acid: A Component of the Inorganic Armamentarium of Innate Immunity. J. Burns Wounds.

[41] Vianna M.E., Gomes B.P.F.A., Berber V.B., Zaia A.A., Ferraz C.C.R., de Souza-Filho F.J. (2004). In Vitro Evaluation of the Antimicrobial Activity of Chlorhexidine and Sodium Hypochlorite. Oral Surg. Oral Med. Oral Pathol. Oral Radiol. Endod..

[42] Radcliffe C.E., Potouridou L., Qureshi R., Habahbeh N., Qualtrough A., Worthington H., Drucker D.B. (2004). Antimicrobial Activity of Varying Concentrations of Sodium Hypochlorite on the Endodontic Microorganisms *Actinomyces israelii, A. naeslundii, Candida albicans* and *Enterococcus faecalis*. Int. Endod. J..

[43] Retamozo B., Shabahang S., Johnson N., Aprecio R.M., Torabinejad M. (2010). Minimum Contact Time and Concentration of Sodium Hypochlorite Required to Eliminate *Enterococcus faecalis*. J. Endod..

[44] Bock L.J., Ferguson P.M., Clarke M., Pumpitakkul V., Wand M.E., Fady P.-E., Allison L., Fleck R.A., Shepherd M.J., Mason A.J. (2021). *Pseudomonas aeruginosa* Adapts to Octenidine via a Combination of Efflux and Membrane Remodelling. Commun. Biol..

[45] Shepherd M.J., Moore G., Wand M.E., Sutton J.M., Bock L.J. (2018). *Pseudomonas aeruginosa* Adapts to Octenidine in the Laboratory and a Simulated Clinical Setting, Leading to Increased Tolerance to Chlorhexidine and Other Biocides. J. Hosp. Infect..

[46] Alsamhary K. (2024). Evaluating the Expression of Efflux Pumps in *Pseudomonas aeruginosa* in Exposure to Sodium Dodecyl Sulfate, Didecyldimethylammonium Chloride, and Octenidine Dihydrochloride. Microb. Drug Resist..

[47] Feßler A.T., Scholtzek A.D., Schug A.R., Kohn B., Weingart C., Hanke D., Schink A.-K., Bethe A., Lübke-Becker A., Schwarz S. (2022). Antimicrobial and Biocide Resistance among Canine and Feline *Enterococcus faecalis*, *Enterococcus faecium, Escherichia coli, Pseudomonas aeruginosa*, and *Acinetobacter baumannii* Isolates from Diagnostic Submissions. Antibiotics.

[48] Rakshit P., Singh A., Singh R., Banerjee T. (2024). An In-Depth Study on Survival Mechanism of Bacterial Isolates in Disinfectants within the Hospital Environment. Front. Cell Infect. Microbiol..

[49] Kampf G. (2016). Acquired Resistance to Chlorhexidine—Is It Time to Establish an ‘Antiseptic Stewardship’ Initiative?. J. Hosp. Infect..

[50] Mombeshora M., Mukanganyama S. (2017). Development of an Accumulation Assay and Evaluation of the Effects of Efflux Pump Inhibitors on the Retention of Chlorhexidine Digluconate in *Pseudomonas aeruginosa* and *Staphylococcus aureus*. BMC Res. Notes.

[51] Wand M.E., Jamshidi S., Bock L.J., Rahman K.M., Sutton J.M. (2019). SmvA Is an Important Efflux Pump for Cationic Biocides in *Klebsiella pneumoniae* and Other Enterobacteriaceae. Sci. Rep..

[52] Monteiro Vasconcelos F., Cabral Pereira da Costa C., Peres E.M., Souza Leão R., Ferraz Gomes H., Silva Thiengo Andrade P.C., Silva Pires A., Faria C., Oliveira Motta A.P., Barreto Pires B.M.F. (2022). Microbiological Identification and Resistance Profile of Microorganisms in Pressure Injuries after the Use of Polyhexamethylene Biguanide: A Series of Fourteen Cases. Wounds.

[53] da Cruz Nizer W.S., Inkovskiy V., Overhage J. (2020). Surviving Reactive Chlorine Stress: Responses of Gram-Negative Bacteria to Hypochlorous Acid. Microorganisms.

[54] Small D.A., Chang W., Toghrol F., Bentley W.E. (2007). Comparative Global Transcription Analysis of Sodium Hypochlorite, Peracetic Acid, and Hydrogen Peroxide on *Pseudomonas aeruginosa*. Appl. Microbiol. Biotechnol..

[55] da Cruz Nizer W.S., Inkovskiy V., Versey Z., Strempel N., Cassol E., Overhage J. (2021). Oxidative Stress Response in *Pseudomonas aeruginosa*. Pathogens.

[56] Hou A.-M., Yang D., Miao J., Shi D.-Y., Yin J., Yang Z.-W., Shen Z.-Q., Wang H.-R., Qiu Z.-G., Liu W.-L. (2019). Chlorine Injury Enhances Antibiotic Resistance in *Pseudomonas aeruginosa* through over Expression of Drug Efflux Pumps. Water Res..

[57] Nontaleerak B., Duang-Nkern J., Wongsaroj L., Trinachartvanit W., Romsang A., Mongkolsuk S. (2020). Roles of RcsA, an AhpD Family Protein, in Reactive Chlorine Stress Resistance and Virulence in *Pseudomonas aeruginosa*. Appl. Environ. Microbiol..

[58] Tong C., Hu H., Chen G., Li Z., Li A., Zhang J. (2021). Chlorine Disinfectants Promote Microbial Resistance in *Pseudomonas* sp.. Environ. Res..

[59] Strempel N., Nusser M., Neidig A., Brenner-Weiss G., Overhage J. (2017). The Oxidative Stress Agent Hypochlorite Stimulates C-Di-GMP Synthesis and Biofilm Formation in *Pseudomonas aeruginosa*. Front. Microbiol..

[60] da Cruz Nizer W.S., Allison K.N., Adams M.E., Vargas M.A., Ahmed D., Beaulieu C., Raju D., Cassol E., Howell P.L., Overhage J. (2024). The Role of Exopolysaccharides Psl and Pel in Resistance of *Pseudomonas aeruginosa* to the Oxidative Stressors Sodium Hypochlorite and Hydrogen Peroxide. Microbiol. Spectr..

